# An Evaluation of a New Autism-Adapted Cognitive Behaviour Therapy Manual for Adolescents with Obsessive–Compulsive Disorder

**DOI:** 10.1007/s10578-020-01066-6

**Published:** 2020-10-06

**Authors:** Amita Jassi, Lorena Fernández de la Cruz, Ailsa Russell, Georgina Krebs

**Affiliations:** 1 OCD, BDD and Related Disorders Clinic, South London and Maudsley NHS Trust, Michael Rutter Centre, De Crespigny Park, London, SE5 8AZ UK; 2 Department of Clinical Neuroscience, Centre for Psychiatry Research, Karolinska Institutet, Stockholm, Sweden; 3 Stockholm Health Care Services, Stockholm County Council, Stockholm, Sweden; 4 Department of Psychology, Centre for Applied Autism Research, University of Bath, Bath, UK; 5 Social, Genetic and Developmental Psychiatry Centre, Institute of Psychiatry, Psychology and Neuroscience, King’s College London, London, UK

**Keywords:** Obsessive–compulsive disorder, Autism spectrum disorder, Treatment outcomes, Cognitive behaviour therapy

## Abstract

Obsessive–compulsive disorder (OCD) and autism spectrum disorder (ASD) frequently co-occur. Standard cognitive behaviour therapy (CBT) for OCD outcomes are poorer in young people with ASD, compared to those without. The aim of this naturalistic study was to evaluate the effectiveness of a novel adolescent autism-adapted CBT manual for OCD in a specialist clinical setting. Additionally, we examined whether treatment gains were maintained at 3-month follow-up. Thirty-four adolescents underwent CBT; at the end of treatment, 51.51% were treatment responders and 21.21% were in remission. At 3-month follow-up, 52.94% were responders and 35.29% remitters. Significant improvements were also observed on a range of secondary measures, including family accommodation and global functioning. This study indicates this adapted package of CBT is associated with significant improvements in OCD outcomes, with superior outcomes to those reported in previous studies. Further investigation of the generalizability of these results, as well as dissemination to different settings, is warranted.

**Electronic supplementary material:**

The online version of this article (10.1007/s10578-020-01066-6) contains supplementary material, which is available to authorized users.

## Introduction

Obsessive–compulsive disorder (OCD) is a common psychiatric condition affecting approximately 0.25–3% of children and adolescents [1, 2]. The disorder causes substantial distress and functional impairment, including poor educational, social, and family functioning [3, 4]. OCD is particularly common among young people with autism spectrum disorder (ASD); it is estimated that up to 37% of those with ASD also experience OCD [5, 6]. Young people with both OCD and ASD (OCD + ASD) have higher levels of functional impairment, utilise more mental health services, and have poorer outcomes following multimodal treatment for OCD, compared to those with OCD who do not have a diagnosis of ASD [7].

Cognitive behaviour therapy (CBT) is the first-line evidence-based treatment for children and adolescents with OCD [8, 9]. Rates of response and remission for children and adolescents with OCD treated with CBT are high [10, 11]. However, previous studies have indicated that young people with OCD + ASD show less improvement in OCD symptoms following standard CBT for OCD both at post-treatment [12] and at 6-month follow-up [13], compared to those who have OCD without ASD. This highlights the need to adapt standard CBT protocols for OCD to better suit the developmental and cognitive profile of young people with OCD + ASD. Research has indicated often the reason why young people with OCD do not respond to CBT is due to ‘technical failures’ i.e. failure due to inadequacy of treatment [14]. Therefore, having a protocol with the key treatment ingredients, and an adequate dose of these ingredients, can reduce the likelihood of technical failures.

A wide range of modifications have been described in single case reports, including extended treatment, greater use of visual aids, providing support with emotion recognition, incorporating special interests, use of idiosyncratic rating scales, and increased parental involvement [15–22]. There have been a number of randomized controlled trials (RCTs) evaluating CBT for anxiety disorders in young people with ASD, which have included some young people with OCD, that have found CBT to be effective for this group [23–25]. Whilst OCD has commonalities with other anxiety disorders, it also regarded as being distinct in its presentation [26, 27] and its treatment [28], with a focus on response prevention as well as exposure to feared stimuli. Therefore, several CBT manuals have been developed specifically for individuals with OCD + ASD which have been evaluated in two RCTs [29, 30] and a case series [31]. The first RCT focused on adults (*n* = 33) and a small number of adolescents aged 14 years and above (*n* = 13) with OCD + ASD and found 20 sessions of modified CBT for OCD to be associated with a higher response rate than anxiety management [29]. The second RCT included only children aged 8–12 years (*n* = 14) and evaluated function-based group CBT targeted at treating obsessive–compulsive behaviours in the broader sense, as opposed to OCD per se [30]. Children randomized to the CBT group showed a significantly greater reduction in obsessive–compulsive behaviours compared to those in the treatment as usual group. Finally, the case series focused on adolescents with OCD + ASD aged 11–17 years (*n* = 9) and evaluated an intensive format of CBT for OCD [31]. Seven of the nine adolescents responded to treatment, which involved a broad range of 24 to 80 (mean = 46.5 ± 20.9) daily CBT sessions. Although this preliminary evidence is encouraging, these studies were generally small [30, 31], focused on symptoms rather than an OCD diagnosis [30], used intensive CBT [31]—which may not be available or feasible to deliver in most clinical settings—or only measured outcomes at post-treatment and did not assess maintenance of gains over time [30, 31].

Further, there are a series of questions that remain unanswered. For example, a widely held clinical view is that individuals with co-occurring OCD and ASD tend to require more OCD treatment sessions over a longer period of time to make meaningful gains, compared to those without ASD. Consistent with this, previous research has shown that young people with OCD + ASD are engaged with clinical services for significantly longer than those without ASD [7]. However, to date, there is no empirical data to support this view, and it remains unclear whether extending treatment confers an added benefit. This is a crucial question as it has important implications for service delivery and resource allocation.

The goal of this study was to evaluate a new CBT manual and workbook specifically developed for adolescents with comorbid OCD and ASD [32, 33] in an open naturalistic study, which tend to have higher external validity than typical RCTs [34]. The treatment manual is based on an existing standard CBT manual for adolescents with OCD [35], but the content has been modified to suit the profile of young people with ASD and the package of treatment has been extended from 14 to 20 weekly sessions [32, 33]. Our study aimed to (i) examine the effectiveness of this new treatment protocol on OCD symptoms, family accommodation, and psychosocial functioning; (ii) establish if the extended treatment duration resulted in additional clinical improvement in OCD symptoms; (iii) assess if the treatment gains were maintained at 3-month follow-up; and, finally, (iv) investigate the general acceptability of the new treatment approach and seek feedback about the ASD-specific components from young people and their parents. We met these aims by delivering the newly modified treatment manual and workbook to a sample of 34 adolescents with OCD + ASD at a specialist clinic.

## Method

### Setting and Study Participants

A total of 34 young people with OCD + ASD were consecutively recruited to the study from January 2015 to March 2018 from referrals to the National and Specialist OCD, BDD and Related Disorders Clinic, South London and Maudsley NHS Trust. The clinic receives referrals from around the UK and often young people have had treatment in their local child and adolescent mental health services before the referral to the specialist team is made.

Initial assessments consisted of a three-hour evaluation by a multi-disciplinary team (see Nakatani et al. [36] for a detailed description of the assessment process). Comorbid diagnoses (except for ASD) were made based on the Development and Well-Being Assessment (DAWBA) [37]. All young people had an established diagnosis of ASD prior to the assessment at the OCD specialist clinic. In the majority of cases (*n* = 23; 67.65%), this ASD diagnostic assessment had involved the Autism Diagnostic Observation Schedule (ADOS) [38] and/or the Autism Diagnostic Interview-Revised (ADI-R) [39]. The remaining 11 young people (32.35%) had been diagnosed with ASD via a clinician assessment without these structured measures. No young people had a diagnosed global learning disability. Participants also completed a series of additional assessment measures (see Measures section).

All data used in the current study were collected as part of clinical practice but are of high standard and routinely employed for research purposes [40, 41]. Study approval was granted by the South London and Maudsley Child and Adolescent Mental Health Service Audit Committee.

### Measures

A series of clinician-reported and self-/parent-reported measures, listed below, were completed. Since effect sizes have been shown to vary for adapted CBT in people with ASD depending on the informant, with smaller effect sizes for self-report and small to medium for informant and clinicians [42], we considered a range of informants. Measures were applied at several time-points, including the initial assessment (baseline), session 7, session 14, end of treatment, and 3-month follow-up (unless otherwise specified).

#### Children’s Yale-Brown Obsessive–Compulsive Scale (CY-BOCS)

The CY-BOCS is a widely used clinician-administered measure of OCD symptoms and severity. It includes a checklist of obsessions and compulsions and a total of 10 items assessing the severity of both obsessions and compulsions (i.e., time spent, interference, distress, resistance, and control), with a total score ranging from 0 to 40. The CY-BOCS has shown excellent psychometric properties with high inter-rater reliability and construct validity [43, 44].

#### Clinical Global Impression Scale—Severity (CGI-S) and Clinical Global Impression Scale—Improvement (CGI-I)

The CGI-S is a clinician rating of symptom severity; ratings range from 1 (normal, not at all ill) to 7 (among the most extremely ill patients) [45]. The CGI-I is a clinician-rated measure of symptom improvement and is also rated on a 7-point scale ranging from 1 (very much improved) to 7 (very much worse) [46]. The CGI-S has demonstrated strong correlations with the CY-BOCS total score (*r* = 0.75) in paediatric OCD research [44]. These scales have been used in research and clinical contexts [47] and has shown good concurrent validity and sensitivity to change [48].

#### Children’s Global Assessment Scale (CGAS)

The CGAS is an adaptation of the Global Assessment Scale for adults. It is a clinician-rated scale that measures global level of functioning in children across different domains. The scale ranges from 1 to 100, with higher scores indicating better functioning. The CGAS has shown good psychometric properties, including good inter-rater reliability [49, 50].

#### The Children’s Obsessive–Compulsive Inventory (ChOCI)

This is a self-report measure assessing severity of OCD symptoms and has both a parent (ChOCI-P) and a child version (ChOCI-C). ChOCI scores range from 0 to 48, with higher scores indicating greater severity. The scale includes an obsessions and a compulsions scale, as well as a total score. The ChOCI has demonstrated to have good internal consistency, criterion validity, and convergent validity [51, 52].

#### Family Accommodation Scale-Parent Report Version (FAS-PR)

This is a 13-item parent report questionnaire that measures the degree to which family members accommodate their child’s OCD symptoms and the level of distress or impairment that they experience as a result [53]. Each item is rated in a 5-point likert scale ranging from 0 (never) to 4 (daily) and enquires about the last month. The scale includes two subscales: involvement in compulsions and avoidance of triggers, as well as a total score [54]. Total scores above 13 indicate clinically significant levels of family accommodation. The FAS-PR has demonstrated a stable factor structure, excellent internal consistency, good convergent validity, and adequate discriminant validity [54].

#### The Repetitive Behaviour Questionnaire (RBQ-2)

This is a 20-item parent report measure developed to assess repetitive behaviours in individuals with ASD, which are a common feature of the disorder. Response choices, based on the last month, are combined into three alternatives for each item (1: never/rarely; 2: mild/occasional; 3: marked/notable). A four-factor model has been proposed as best fit for the measure, including repetitive motor movements, rigidity/adherence to routine, preoccupations with restricted patterns of interest, and unusual sensory interests [55]. The RBQ-2 has good internal consistency and inter-item validity [55]. This measure was applied at all time-points except for session 14. Since scores on the RBQ-2 were not expected to change over time (since the applied OCD treatment does not target ASD symptoms), it was not considered an outcome measure as such.

#### The Work and Social Adjustment Scale-Youth Version (WSAS-Y) and Parent Version (WSAS-P)

This is a self-report measure assessing functional impairment resulting from the presenting condition (i.e., OCD). The original version for adults was developed by Marks [56] and it has been adapted for use in young people and their parents [57]. It consists of five items assessing global impairment, with scores ranging from 0 to 40, with higher scores indicating greater functional impairment. The WSAS-Y/P has demonstrated to have excellent internal consistency, adequate test–retest reliability, and good convergent validity [57].

#### Treatment Satisfaction Survey

Young people and families were given a series of questions (see **Supplement**) asking how overall satisfied they were with the received treatment, as well as with specific components of the treatment, including the ASD modifications (e.g., visual materials and worksheets, sessions on understanding ASD and the differences between OCD and ASD, and parental involvement in the sessions). The satisfaction survey was only applied at post-treatment.

### Modified CBT for OCD in Youth with ASD

All participants received individual modified CBT that included as a main component exposure and response prevention (ERP) [32, 33] delivered by experienced clinical psychologists, all of whom had a doctoral level of clinical training, who specialised in the treatment of OCD and had between 2–16 years of experience with this patient group. All therapists attended a training workshop delivered by author AJ which covered what was included in the adapted treatment protocol. Each therapist received weekly supervision from a senior clinical psychologist and was encouraged to bring recordings of sessions for discussion to the supervision sessions. The modified CBT protocol was an adaptation of a standard CBT protocol for OCD [35]. While the original protocol involves 14 sessions, treatment in this study was extended to include 20 sessions, which were delivered weekly. Details of the treatment and specific ASD modifications are outlined in Table 1.

**Table 1:** Description of cognitive behaviour therapy for obsessive–compulsive disorder, with autism spectrum disorder modifications

	Session content	ASD modifications
Sessions 1–6	**Psychoeducation on OCD, ASD, and anxiety** Externalising OCD, normalising anxiety, reframing anxiety as a protective mechanism (the ‘fight or flight’ response), and OCD hierarchy formation	Differentiating OCD- and ASD-related repetitive behaviour. Extended psychoeducation on anxiety. Understanding how anxiety differs from other emotions. Development of an idiosyncratic anxiety rating scale.
Sessions 7–19	**Graded ERP** Young people facing increasingly challenging fears on an OCD hierarchy	Visual, mini-hierarchies for each step of the main hierarchy to allow young people to take smaller steps during the exposure process. Emphasising the similarities between tasks conducted in sessions and for homework to promote generalisation. Off-site visits to conduct ERP tasks to make them ecologically valid. Families leading ERP tasks to allow them to be able to use the tools between sessions and to prepare them for when treatment ends.
Session 20	**Relapse prevention** Reflecting on progress in treatment. Reviewing goals set at the beginning of treatment. Developing a plan of what to do in the event of a set-back. Setting goals for the future.	

*ASD* Autism Spectrum Disorder, *ERP* Exposure and Response Prevention, *OCD* Obsessive–Compulsive Disorder

All participants progressed through the treatment manual in the same order. The psychoeducation phase of this modified treatment took up to six sessions, whereas in the standard package this typically takes two sessions [35]. Other broader ASD modifications of the standard protocol were the highly structured content of each session, with timings being put on agenda items and being ticked off as session progressed, visual worksheets, and incorporation of special interests wherever possible. Scheduled homework each week was set to consolidate in-session learning. Parents were encouraged to attend sessions, either by sitting in for the entire session or joining for a parent check-in at the end of the session to hear what was covered and what homework was set. Sessions took place in the clinic, at home, and in environments where OCD typically got triggered.

### Statistical Analysis

Mixed-effects regression analyses for repeated measures with maximum likelihood estimation (MLE) of parameters were implemented in Stata SE/13.1. Mixed-effects models use all available data, can properly account for correlation between repeated measurements on the same subject, have greater flexibility to model time effects, and can handle missing data [58]. For each outcome measure, the model included fixed effects of time and subject effects as a random intercept factor to account for the variances between participants and within participants.

Additionally, percentages of treatment responders and remitters were calculated at the end of the treatment and at 3-month follow-up. According to consensus definitions [59], response was defined as a reduction ≥ 35% in the CY-BOCS score and a CGI-I score of 1 or 2, and remission was defined as a score of ≤ 12 in the CY-BOCS and a CGI-S of 1 or 2. Alpha (two-tailed) was set at p < 0.05 for all analyses. Numbers may vary across analyses as a result of missing values.

## Results

### Demographic and Baseline Clinical Characteristics of the Sample

Demographic and clinical characteristics of the 34 study participants are summarized in Table 2. Two thirds of the sample were boys (67.64%), the mean age at assessment was approximately 15 years (range 11–17), and the mean age of OCD onset was 11 years. A very large majority (*n* = 31; 91%) were on psychotropic medication at the time of the initial assessment. All patients on medication were on selective serotonin reuptake inhibitors. Additionally, seven of these individuals were on antipsychotics and two were on other drugs, namely procyclidine, lithium, and attention-deficit/hyperactivity disorder drugs. Twenty-five (73.52%) had previously undertaken CBT treatment at the time of the initial assessment. Seven (20.58%) participants met diagnostic criteria for at least one other psychiatric disorder, besides OCD and ASD, most commonly an anxiety disorder.

**Table 2:** Baseline demographic and clinical characteristics of the sample of children and adolescents with obsessive–compulsive disorder and comorbid autism spectrum disorder (*n* = 34)

Demographic variables	Data available *n*	Frequency	%	
Sex, boys	34	23	67.64	–

Demographic variables	Data available *n*	Mean	sd	Range
Age at assessment, in years	34	15.18	1.70	11–17
Age of onset of the OCD, in years	29	11.52	2.68	7–16

Clinical variables	Data available *n*	Frequency	%	
On medication at the time of assessment	34	31	91.18	–
Previous cognitive behaviour therapy	34	25	73.53	–
Psychiatric comorbidity, besides OCD and ASD	34	7	20.58	–
Anxiety disorders	34	4	11.76	–
Attention-deficit/hyperactivity disorder	34	2	5.88	–
Body dysmorphic disorder	34	1	2.94	–
Tourette syndrome	34	1	2.94	–

Psychiatric symptoms measures	Data available *n*	Mean	sd	Range
*Clinician-reported*				
CY-BOCS	34	27.65	4.15	15–34
CGI-S	34	5.06	0.89	3–6
CGAS	33	42.24	7.94	27–63
*Self- and parent-reported*				
CHOCI-C total	20	30.80	8.71	15–45
CHOCI-C obsessions	20	14.80	5.32	0–23
CHOCI-C compulsions	20	16.00	4.30	8–22
CHOCI-P total	24	32.62	9.25	0–44
CHOCI-P obsessions	25	16.28	4.81	0–22
CHOCI-P compulsions	24	16.46	5.16	0–22
FAS-PR total	24	29.67	13.67	3–47
FAS-PR avoidance	24	15.08	7.47	0–24
FAS-PR involvement	24	14.58	7.00	3–23
RBQ-2 total	18	38.28	9.36	22–53
RBQ-2 repetitive motor movements	18	9.00	3.66	5–17
RBQ-2 rigidity/adherence to routine	18	14.50	4.45	0–20
RBQ-2 preoccupation/restricted patterns	18	12.33	3.36	7–19
RBQ-2 unusual sensory interests	18	7.44	2.87	3–14
WSAS-Y	18	20.61	11.11	5–40
WSAS-P	21	27.52	7.63	14–40

*ASD* Autism Spectrum Disorder, *CGAS* Children’s Global Assessment Scale, *CGI-S* Clinical Global Impression—Severity, *CHOCI-C* Children’s Obsessional Compulsive Inventory—Child Version, *CHOCI-P* Children’s Obsessional Compulsive Inventory—Parent Version, *CY-BOCS* Children’s Yale-Brown Obsessive Compulsive Scale, *FAS* Family Accommodation Scale, *OCD* Obsessive–Compulsive Disorder, *RBQ* Repetitive Behaviours Questionnaire, *WSAS-P* Work and Social Adjustment Scale—Parent Version, *WSAS-Y* Work and Social Adjustment Scale—Youth Version

Youth in the sample showed moderate to severe symptoms of OCD, as measured with the CY-BOCS, the ChOCI-C, and the ChOCI-P. Additionally, they were assessed as markedly ill, according to the CGI-S. Mean FAS-PR scores fell well above the clinical cut-off of 13, indicating clinically significant levels of family accommodation. Participants presented scores in the moderate degree of interference in functioning in all domains, according to the CGAS. This impairment was also reflected in the high scores on the WSAS-Y and WSAS-P.

### Treatment Outcomes Following CBT for OCD in Youth with ASD

**Fig. 1:**
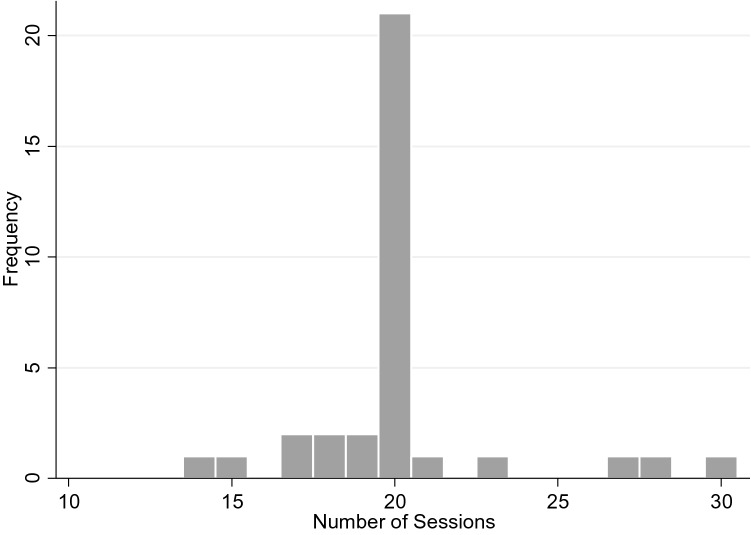
Histogram for the number of sessions received during the treatment by the study participants (*n* = 34)

Young people in the study received a mean number of 20 sessions (*sd* = 3.07, range 14–30; Fig. 1). Despite the treatment was protocolised and had a standard duration of 20 sessions, the naturalistic nature of the study and the fact that patients were receiving this treatment at a regular specialist clinic allowed for a certain degree of flexibility. Five study participants received less than the 20 stipulated sessions (two dropped out of treatment after sessions 14 and 15 and three completed treatment at sessions 17, 18, and 19 due to an improvement of their symptoms) and five more received 21, 23, 27, 28, and 30 sessions since the corresponding therapist considered that further benefit could be obtained from those extra sessions before terminating the treatment.

**Fig. 2:**
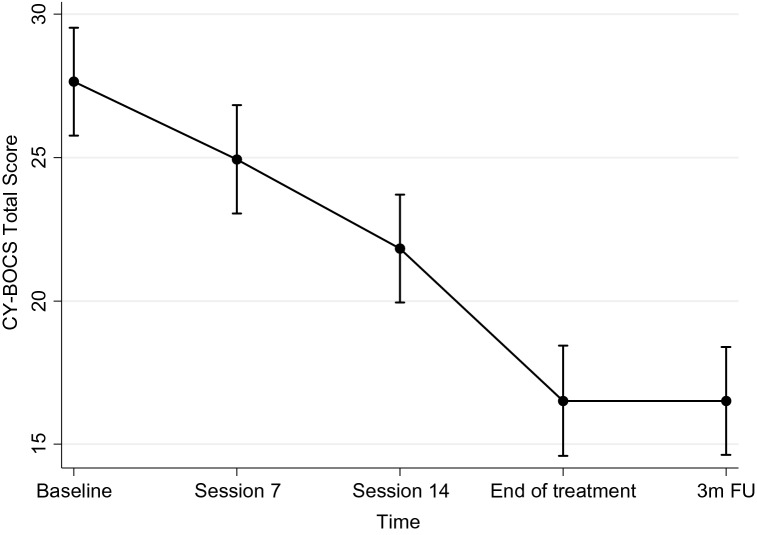
Time effects on the Children’s Yale-Brown Obsessive–Compulsive Scale, derived from the mixed-effects regression model. Error bars indicate 95% confidence intervals. *C-BOCS* Children’s Yale-Brown Obsessive Compulsive Scale, *3 m FU* 3-month follow-up

Estimated mean CY-BOCS scores and standard errors (SE) from the mixed-effects model for each time-point are shown in Table 3. The linear mixed-effect regression model on the CY-BOCS revealed that there was a significant effect of time at all time-points, compared to baseline, indicating an improvement of the OCD symptoms over time (Table 4 and Fig. 2). The estimated reduction from baseline to post-treatment was around 11 points in the CY-BOCS (− 11.14 [− 13.07, − 9.20]).

**Table 3:** Model estimates across time-points for each outcome measure from the linear mixed-effects models

Measures of psychiatric symptoms	Baseline		Session 7		Session 14		End of treatment		3-month follow-up	
	Mean	SE	Mean	SE	Mean	SE	Mean	SE	Mean	SE
CY-BOCS	27.65	0.96	24.94	0.96	21.82	0.96	16.51	0.98	16.50	0.96
CGAS	42.16	1.66	45.10	1.71	49.57	1.71	53.82	1.64	54.75	1.66
CHOCI-C total	30.45	2.06	23.04	1.86	25.21	2.36	17.28	2.51	19.69	2.43
CHOCI-C obsessions	14.52	1.16	9.29	1.04	11.85	1.33	10.29	1.42	9.51	1.37
CHOCI-C compulsions	15.71	1.08	13.76	0.99	13.36	1.22	10.09	1.30	10.31	1.26
CHOCI-P total	32.53	2.11	24.63	2.03	24.06	2.64	19.11	2.49	17.78	2.83
CHOCI-P obsessions	16.24	1.14	10.51	1.12	11.94	1.50	9.31	1.37	8.33	1.56
CHOCI-P compulsions	16.43	1.10	14.11	1.06	12.71	1.38	9.77	1.30	9.52	1.48
FAS total	28.85	2.34	27.89	2.37	22.75	2.60	17.84	2.55	17.25	2.64
FAS avoidance	14.80	1.26	13.39	1.28	10.58	1.40	8.33	1.38	8.19	1.43
FAS involvement	14.06	1.23	14.48	1.25	12.16	1.37	9.49	1.35	9.04	1.39
RBQ-2 total^a^	37.75	2.35	31.79	2.22	–	–	29.81	2.53	30.29	2.69
RBQ-2 repetitive motor Movements	8.90	0.70	6.92	0.66	–	–	5.91	0.73	6.28	0.80
RBQ-2 rigidity/adherence to Routine	14.30	1.10	13.13	1.04	–	–	11.65	1.19	12.56	1.27
RBQ-2 preoccupation/restricted Patterns	12.23	0.78	9.99	0.74	–	–	9.32	0.84	9.48	0.89
RBQ-2 unusual sensory interests	7.26	0.53	5.68	0.50	–	–	5.76	0.56	5.65	0.59
WSAS-Y	20.62	2.04	20.62	1.98	21.12	2.23	15.54	2.22	13.23	2.28
WSAS-P	27.13	1.72	23.68	1.73	20.16	1.92	17.11	1.88	18.05	1.97

*CGAS* children’s global assessment scale, *CHOCI-C* children’s obsessional compulsive inventory—child version, *CHOCI-P* children’s obsessional compulsive inventory—parent version, *CY-BOCS* children’s Yale-brown obsessive compulsive scale, *FAS* family accommodation scale, *RBQ* repetitive behaviours questionnaire, *WSAS-P* work and social adjustment scale—parent version, *WSAS-Y* work and social adjustment scale—youth version

^a^The RBQ-2 was not applied at session 14

**Table 4:** Results of the time effects across time-points for each outcome measure from the linear mixed-effects models

Measures of psychiatric symptoms	Coefficient (95% CIs)			
	Baseline to session 7 outcomes	Baseline to session 14 outcomes	Baseline to end of the treatment outcomes	Baseline to 3-month follow-up outcomes
CY-BOCS	− 2.71 (− 4.61, − 0.81)**	− 5.82 (− 7.72, − 3.92)***	− 11.14 (− 13.07, − 9.20)***	− 11.16 (− 13.05, − 9.25)***
CGAS	2.94 (− 0.56, 6.43)	7.40 (3.90, 10.90)***	11.66 (8.29, 15.02)***	12.58 (9.19, 15.97)***
CHOCI-C total	− 7.41 (− 12.29, − 2.54)**	− 5.23 (− 10.91, 0.44)	− 13.17 (− 19.04, − 7.29)***	− 10.76 (− 16.52, − 5.00)***
CHOCI-C obsessions	− 5.23 (− 8.04, − 2.41)***	− 2.67 (− 5.93, 0.59)	− 4.22 (− 7.61, − 0.84)*	− 5.00 (− 8.32, − 1.69)**
CHOCI-C compulsions	− 1.95 (− 4.33, 0.43)	− 2.35 (− 5.15, 0.45)	− 5.62 (− 8.50, − 2.74)***	− 5.40 (− 8.24, − 2.57)***
CHOCI-P total	− 7.90 (− 13.19, − 2.60)**	− 8.47 (− 14.73, − 2.21)**	− 13.42 (− 19.40, − 7.44)***	− 14.75 (− 21.27, − 8.22)***
CHOCI-P obsessions	− 5.72 (− 8.60, − 2.85)***	− 4.29 (− 7.79, − 0.80)*	− 6.92 (− 10.18, − 3.67)***	− 7.91 (− 11.48, − 4.34)***
CHOCI-P compulsions	− 2.32 (− 5.08, 0.44)	− 3.72 (− 6.98, − 0.45)*	− 6.66 (− 9.78, − 3.55)***	− 6.90 (− 10.31, − 3.50)***
FAS total	− 0.96 (− 5.04, 3.12)	− 6.10 (− 10.69, − 1.51)**	− 11.00 (− 15.40, − 6.61)***	11.60 (− 16.19, − 7.01)***
FAS avoidance	− 1.41 (− 3.62, 0.80)	− 4.22 (− 6.71, − 1.74)**	− 6.47 (− 8.85, − 4.10)***	− 6.61 (− 9.09, − 4.12)***
FAS involvement	0.42 (− 1.78, 2.62)	− 1.89 (− 4.37, 0.58)	− 4.56 (− 6.93, − 2.20)***	− 5.01 (− 7.49, − 2.54)***
RBQ-2 total^a^	− 5.96 (− 11.19, − 0.73)*	–	− 7.93 (− 13.67, − 2.20)**	− 7.45 (− 13.52, − 1.39)*
RBQ-2 repetitive motor Movements	− 1.98 (− 3.57, − 0.38)*	–	− 2.99 (− 4.70, − 1.29)**	− 2.62 (− 4.46, − 0.78)**
RBQ-2 rigidity/adherence to Routine	− 1.17 (− 3.67, 1.34)	–	− 2.65 (− 5.40, 0.09)	− 1.73 (− 4.64, 1.17)
RBQ-2 preoccupation/restricted Patterns	− 2.24 (− 3.91, − 0.58)**	–	− 2.91 (− 4.73, − 1.09)**	− 2.75 (− 4.68, − 0.82)**
RBQ-2 unusual sensory interests	− 1.59 (− 2.62, − 0.55)**	–	− 1.50 (− 2.63, − 0.37)**	− 1.61 (− 2.82, − 0.41)**
WSAS-Y	0.00 (− 4.09, 4.10)	0.50 (− 4.12, 5.13)	− 5.08 (− 9.65, − 0.50)*	− 7.38 (− 12.08, − 2.68)**
WSAS-P	− 3.45 (− 7.33, 0.43)	− 6.98 (− 11.14, − 2.81)**	− 10.02 (− 14.06, − 5.98)***	− 9.08 (− 13.30, − 4.87)***

*CGAS* Children’s Global Assessment Scale, *CHOCI-C* Children’s Obsessional Compulsive Inventory—Child Version, *CHOCI-P* Children’s Obsessional Compulsive Inventory—Parent Version, *CY-BOCS* Children’s Yale-Brown Obsessive Compulsive Scale, *FAS* Family Accommodation Scale, *RBQ* Repetitive Behaviours Questionnaire, *WSAS-P* Work and Social Adjustment Scale—Parent Version, *WSAS-Y* Work and Social Adjustment Scale—Youth Version

^a^The RBQ-2 was not applied at session 14

^***^p < 0.001 **p < 0.01 *p < 0.05

An additional pairwise comparison between session 14 and the end of the treatment showed a notable, statistically significant reduction of the estimate between these time-points (− 5.31 [− 7.25, − 3.38]), which indicated that the addition of extra sessions to the regular 14-session protocol translated into further significant OCD symptom improvement.

In order to assess durability of the CY-BOCS reduction, we ran a pairwise comparison between the end of the treatment time-point and the 3-month follow-up time-point, which resulted in a non-significant estimate (0.01 [− 1.95, 1.92], p = 0.991), indicating that the OCD symptoms remained stable during the follow-up, after treatment termination.

Results for the rest of measures for the study participants are also shown in Tables 3 and 4. The results of the linear mixed-effect regression models for these outcomes showed overall significant improvements across all measures to both the end of the treatment and the 3-month follow-up, relative to baseline. Whilst the RBQ-2 was not considered an outcome measure per se, the total scores and all subscales, with the only exception being Rigidity/Adherence to Routine subscale, also showed a significant improvement over time.

### Treatment Response and Remission

The mean CY-BOCS percentage reduction from baseline to post-treatment was 38%, while the percentage reduction from baseline to the 3-month follow-up was 40%. Based on these reductions and on the CGI-I scores, 17 out of 33 participants (51.51% – one excluded due to missing CGI-S score) were classified as treatment responders at the end of the treatment and 18 individuals out of 34 participants (52.94%), were responders at the 3-month follow-up. Similarly, based on CY-BOCS and CGI-S scores, seven participants out of 33 (21.21%—one excluded due to missing CGI-I score) were classified as remitters at the end of the treatment; this number increased to 12 out of 34 (35.29%) at the 3-month follow-up.

### Treatment Satisfaction

Sixteen of the 34 (47.05%) young people and 18 parents (52.94%) responded to the survey. A total of 13 (81.25%) young people and 18 (100%) parents reported that they were overall either very happy or happy with the treatment they received. A majority said they found CBT taught them many or some useful techniques for fighting OCD (*n* = 14; 87.5% of young people and *n* = 17; 94.44% of parents). All ASD-treatment modification elements were rated as very helpful or somewhat helpful by the majority of young people and parents: visual material (*n* = 13; 81.25% of young people and *n* = 15; 83.33% of parents); learning about the differences between OCD and ASD (*n* = 13; 81.25% of young people and *n* = 16; 88.89% of parents); learning about anxiety and differentiating anxiety from other emotions (*n* = 12; 81.25% of young people and *n* = 16; 88.89% of parents); involvement of family in sessions (*n* = 12; 75% of young people and *n* = 18; 100% of parents); and graded exposure during sessions and for homework (*n* = 13; 81.35% of young people and *n* = 18; 100% of parents).

## Discussion

The current study represents the largest evaluation to date of manualised autism-adapted CBT for adolescents with OCD. We successfully delivered a modified treatment programme which was well received by young people and their parents. We found that OCD symptoms significantly reduced over the course of treatment, with gains being maintained at 3-month follow-up. At the end of treatment, approximately half of the adolescents could be classified as OCD treatment responders, and just over a fifth as remitters. The response rate is broadly similar to that in a previous study of standard CBT for OCD (14 sessions) in children and adolescents with ASD carried out by our group [9] (51.51% in the current study *vs.* 46% in the previous study), while the rates of remission in our study were higher (21.21% in the current study *vs.* 9% in the previous study). This discrepancy is unlikely to be explained by differences in the samples between these studies; both groups were treated in the same specialist clinic, had high-moderate OCD severity, similar proportions were on SSRI medication, and were comparable in age and age of onset of OCD. Moreover, the response and remission criteria employed in the current study were more stringent, with the inclusion of the CGI-I and CGIS-S and not just relying on CY-BOCS reduction or score as a measure [59]. Thus, our findings provide preliminary evidence that the modified CBT package is likely to be associated with superior outcomes when treating young people with ASD, compared to standard CBT for OCD.

Previous studies have recommended an extended treatment programme for people with comorbid OCD + ASD [29]. The current study was the first to examine if this is of added benefit. We found significant reductions in OCD symptoms during the extended phase of treatment (i.e., beyond the standard 14 sessions). There was a decrease of around 5 points in the CY-BOCS score from session 14 to end of treatment, which was similar to the reduction obtained between baseline and session 14. This indicates that the additional sessions, where there is a main focus on ERP, significantly contributed to a further improvement of the OCD symptoms. Moreover, young people and their families were overall satisfied with the treatment and noted that they found the ASD-specific modifications to be beneficial.

A strength of this study was the inclusion of a range of secondary outcome measures. Evidence of improvement over time in OCD symptoms was evident across informants. Specifically, parent- and child-reported OCD symptom measures mirrored that of clinicians, with significant reductions in total scores, already evident by session seven. It is interesting that OCD symptoms significantly reduced from baseline to session seven, and in particular the self- and parent-reported levels of obsessions, rather than compulsions. Our hypothesis is that the longer psychoeducation received as first element of the modified treatment may have offered support to young people with ASD in understanding and feeling less distressed about their obsessions. Of interest, significant reductions in family accommodation were found by the end of treatment and at 3-month follow-up. However, reductions were not appreciated at session seven. This is in keeping with treatment content, whereby family accommodation is only addressed from session seven onwards within an ERP framework [32, 33].

Whilst ASD-related repetitive behaviours were not specifically targeted in treatment or deemed an outcome measure as such, it was interesting to see that there was a significant reduction by the end of treatment in repetitive motor movements, unusual sensory interests, and preoccupations with restricted patterns of interest. However, there was no significant change in rigidity/adherence to routine. It may be the case that study participants were able to apply some of the principles of CBT for OCD to some repetitive behaviour domains. Alternatively, construct overlap in measurement methods mean that the RBQ-2 may be capturing OCD-related compulsions which were explicit treatment targets. Then again, a more general reduction in anxiety levels brought about by OCD-related improvements may confer wider benefits in respect of autism symptoms. Significant associations between several subscales of the repetitive behaviour measure used in the present study and anxiety have been reported, with researchers proposing a complex mediating relationship between sensory hypo- and hyper-reactivity and anxiety [60].

The results of this study have several clinical implications for future research and for the planning of clinical services and policies. We have shown the preliminary effectiveness of an intervention delivered following a workbook and manual [32, 33], with weekly supervision, indicating the potential for this treatment to be transferable and benefit a large proportion of adolescents with OCD + ASD. Future work in larger samples should focus on investigating whether this programme generalises to different contexts and populations with the final goal of disseminating it broadly to different settings, such as non-specialist clinics. We have shown the value of modifying a treatment protocol by adding ASD-tailored elements and additional sessions. If the improvement in treatment outcomes using this tailored programme is indeed confirmed to be superior to the improvement obtained with the standard programme in a RCT, the use of the modified treatment could translate into a reduction of clinical and societal costs by reducing clinical contacts and other costs derived from patient-related impairment such as school absence or parental leave.

Our results should also be considered in the context of some limitations. First, whilst this study is larger than most conducted to date (previous sample sizes ranged from 9 to 25 participants) [12, 13, 30, 31], this study still had a relatively small sample size. Second, given that this was a naturalistic study, there was some degree of data loss. However, this mainly applied to secondary self-reported and parent-reported measures. For the CY-BOCS, for example, which was our main outcome, only two out of 170 possible data points (34 participants by 5 time-points) were missing, corresponding to the end of the treatment (session 20) scores of two individuals that dropped out of treatment after sessions 14 and 15 despite their lack of improvement, although the 3-month follow-up measures could be gathered for both. Third, given that we did not have a waitlist control group, we cannot be sure that symptoms did not spontaneously improve with the passage of time. However, this is unlikely given that this group had had OCD for an average of four years and a vast majority had had previous CBT for the disorder (74%) and/or were already on SSRI medication (91%) without having shown a satisfactory treatment response. Additionally, this study did not have the benefit of an active control condition to assess whether modified CBT was superior to other treatments, including standard CBT for OCD. Despite comparing our figures to those reported in a previous study conducted in the same population in our clinic, a head to head comparison under the same conditions would be necessary. Fourth, whilst we used a protocolised treatment and all therapists were experienced and closely supervised, we did not take formal measures of protocol adherence. Finally, the protocol was tested at a specialist OCD and related disorders clinic and it whether results would generalize to other settings remains to be tested.

## Summary

This study showed that a protocolised CBT for OCD package systematically incorporating modifications for adolescents with ASD was associated with significant improvements in OCD symptoms as well as family accommodation and psychosocial functioning. Against expectations, there were also changes in ASD-related repetitive behaviours throughout the course of treatment. Treatment gains after 14 sessions were further maximised at session 20. Additionally, treatment outcomes were durable up to the 3-month follow-up time-point. Young people and parents receiving the treatment were overall satisfied and highlighted the benefit of specific modifications for ASD. Further investigation of the generalizability of these treatment results, as well as dissemination to different settings, is warranted.

## Electronic supplementary material

Below is the link to the electronic supplementary material.

Supplementary file1 (DOCX 15 kb)
