# Supplementary material for: An Evaluation of a New Autism-Adapted Cognitive Behaviour Therapy Manual for Adolescents with Obsessive–Compulsive Disorder
Source: Child Psychiatry Hum Dev. 2020 Oct 6;52(5):916–27. doi: 10.1007/s10578-020-01066-6 (PMC8405512; doi:10.1007/s10578-020-01066-6)
Supplement: Supplementary file 1 — Supplementary file1 (DOCX 15 kb) [file 10578_2020_1066_MOESM1_ESM.docx]

**Supplement – Treatment satisfaction questions for young people/parents.**

1. How happy were you with the type of CBT you received?

- Very happy
- Happy
- Neutral
- Not happy
- Very unhappy

1. Do you think CBT taught you useful techniques for fighting OCD?

- Many useful techniques
- Some useful techniques
- Neutral
- Not very many useful techniques
- No useful techniques

1. How useful did you find the following parts of CBT?

(Rated very useful, somewhat useful, neutral, not very useful or not useful at all)

- Visual materials
- Learning about differences between ASD and OCD
- Learning about anxiety and other emotions
- Exposure tasks in sessions
- Exposure tasks at home
- Parents coming to sessions
